# The *TERT* rs2736100 polymorphism increases cancer risk: A meta-analysis

**DOI:** 10.18632/oncotarget.16309

**Published:** 2017-03-17

**Authors:** Hui Li, Yanyan Xu, Hua Mei, Liang Peng, Xiaojie Li, Jianzhou Tang

**Affiliations:** ^1^ Department of Microbiology and Immunology, Medical School of Jishou University, Jishou 416000, Hunan, China; ^2^ Department of Molecular Pathology, Guangzhou Kingmed Center for Clinical Laboratory, Guangzhou 510000, China; ^3^ Department of Somatic Stem Cell, Hunan Guangxiu Hospital, Changsha 410002, Hunan, China; ^4^ Department of Biological and Environmental Engineering, Changsha University, Changsha 410003, Hunan, China; ^5^ College of Animal Science and Technology of Hunan Agriculture University, Changsha 410128, Hunan, China

**Keywords:** *TERT*, cancer, risk, meta-analysis, telomerase

## Abstract

Abnormal telomerase activity is implicated in cancer initiation and development. The rs2736100 T > G polymorphism in the telomerase reverse transcriptase (*TERT*) gene, which encodes the telomerase catalytic subunit, has been associated with increased cancer risk. We conducted a meta-analysis to more precisely assess this association. After a comprehensive literature search of the PubMed and EMBASE databases up to November 1, 2016, 61 articles with 72 studies comprising 108,248 cases and 161,472 controls were included in our meta-analysis. Studies were conducted on various cancer types. The *TERT* rs2736100 polymorphism was associated with increased overall cancer risk in five genetic models [homozygous model (GG vs. TT): odds ratio (OR) = 1.39, 95% confidence interval (95% CI) = 1.26-1.54, *P* < 0.001; heterozygous model (TG vs. TT): OR = 1.16, 95% CI = 1.11-1.23, *P* < 0.001; dominant model (TG + GG vs. TT): OR = 1.23, 95% CI = 1.15-1.31, *P* < 0.001; recessive model (GG vs. TG + TT): OR = 1.25, 95% CI = 1.16-1.35, *P* < 0.001; and allele contrast model (G vs. T): OR = 1.17, 95% CI = 1.12-1.23, *P* < 0.001]. A stratified analysis based on cancer type associated the polymorphism with elevated risk of thyroid cancer, bladder cancer, lung cancer, glioma, myeloproliferative neoplasms, and acute myeloid leukemia. Our results confirm that the *TERT* rs2736100 polymorphism confers increased overall cancer risk.

## INTRODUCTION

Cancer is a major public health problem worldwide, with an estimated 14.1 million new cancer cases and 8.2 million deaths in 2012 [1]. Carcinogenesis is a complex process, influenced by various genetic and environmental factors, such as smoking, poor diet, physical inactivity, reproductive changes and the growth and aging of the population [1, 2]. Telomeres, composed of the TTAGGG repeat sequence, are special chromatin structures located at each end of a chromosome. Telomeres maintain chromosomal integrity by protecting chromosome ends from DNA damage and end-to-end fusions [3]. Abnormally short telomeres may cause chromosomal instability, and consequentially contribute to cancer development. Telomerase (also known as terminal transferase), a reverse transcriptase enzyme, extends the 3′ end of chromosomal DNA by catalyzing the telomere synthesis reaction. Defects in telomerase activity have been observed in many human tumor cells, and telomere length was inversely associated with cancer incidence and mortality [4]. Telomerase reverse transcriptase (TERT), the telomerase catalytic subunit, maintains telomere stability [5]. In a previous genome-wide association study (GWAS), Shete, *et al*. discovered that certain *TERT* gene variants increase glioma susceptibility [6]. Since then, *TERT* variants have been associated with various cancers, including breast, lung, colorectal, ovarian, prostate, and gastric cancers [7, 8].

The *TERT* gene is located in 5p15.33. The rs2736100 T > G polymorphism in the second intron of the *TERT* gene has been associated with shortened telomere length in gastric cancer [9]. The association of this SNP with cancer susceptibility has been extensively explored, although the findings are as yet inconclusive. Several meta-analyses published in 2014 associated the *TERT* rs2736100 polymorphism with increased glioma and lung cancer susceptibility [10–14]. In 2012, Zou, *et al*. observed an association between this polymorphism and overall cancer risk [15], although their meta-analysis involved only 11 articles. However, between 2015 and 2016, more than 27 studies were published with large sample sizes [9, 16–37]. Thus, we performed an updated meta-analysis to more precisely assess the *TERT* rs2736100 polymorphism-cancer association, including 72 studies derived from 61 articles with 269,720 total subjects [6, 9, 16–74].

## RESULTS

### Study characteristics

**Figure 1:**
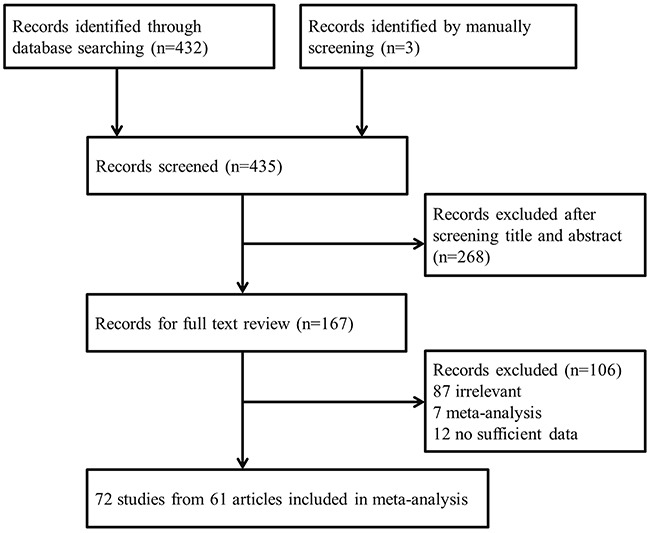
Flowchart of articles included in our meta-analysis

We initially identified 432 records from the PubMed and EMBASE databases (Figure 1). After screening titles and abstracts, 268 articles were excluded and the full texts of the remaining 164 articles were further assessed. Articles were excluded for the following reasons: irrelevant association (87 articles), meta-analysis (7), and lacking sufficient raw data for further evaluation (12). Three additional articles were identified by manually screening the references of relevant articles. Finally, 72 studies extracted from 61 articles met our study inclusion criteria and were included in the current meta-analysis [6, 9, 16–74].

In most of the included studies, the *TERT* rs2736100 polymorphism genotypic distribution followed Hardy-Weinberg equilibrium (HWE) in controls, except for seven studies [6, 28, 43, 51, 63, 66, 72]. Since the genotype distributions of other polymorphisms were in compliance with HWE in these seven studies, we included these studies in the meta-analysis. In total, 72 studies with 108,248 cases and 161,472 controls were included in our pooled analysis. Studies were conducted on various cancer types, including lung (28 studies), glioma (5), colorectal (4), bladder (4), myeloproliferative neoplasms (MPN) (4), gastric (3), acute myeloid leukemia (AML) (2), breast (2), melanoma (2), and thyroid (2). The remaining 16 studies focused on different types of cancer, with one study for each type of cancer, and were grouped together as “other cancer” in our analyses. There were 37 studies conducted in Asians and 35 in Caucasians. Twenty-three studies included fewer than 500 controls, and 49 had 500 or more controls. Sixteen studies were categorized as low quality and 56 were high quality. The main characteristics of all the studies are summarized in Table 1.

**Table 1:** The main characteristics of all the studies included in the meta-analysis

Surname	Year	Country	Ethnicity	Cancer type	Cases	Controls							HWE	Score
					All	TT	TG	GG	All	TT	TG	GG		
Zhou	2016	China	Asian	ESCC	588	165	275	148	600	215	287	108	0.472	11
Zhang	2016	China	Asian	NC	855	265	428	162	1036	365	516	155	0.211	13
Yuan	2016	China	Asian	UTUC	212	83	81	48	289	86	144	59	0.928	10
Xing	2016	China	Asian	Lung cancer	418	216	164	38	410	268	124	18	0.452	10
Wang	2016	China	Asian	Lung cancer	500	131	257	112	500	178	242	80	0.881	11
Trifa	2016	Romania	Caucasian	MPN	529	76	255	198	433	124	213	96	0.802	13
Krahling 1	2016	Hungary	Caucasian	PMN	584	77	282	225	400	111	188	101	0.235	8
Krahling 2	2016	Hungary	Caucasian	CML	86	25	43	18	400	111	188	101	0.235	8
Krahling 3	2016	Hungary	Caucasian	AML	308	71	153	84	400	111	188	101	0.235	7
Gong	2016	China	Asian	Thyroid cancer	452	142	214	96	452	156	222	74	0.738	11
Ge	2016	China	Asian	Thyroid cancer	2300	644	1093	563	2300	875	1056	369	0.093	12
Dahlstrom 1	2016	Sweden	Caucasian	MPN	126	15	64	47	756	167	377	212	0.980	9
Dahlstrom 2	2016	China	Asian	MPN	101	17	52	32	101	33	50	18	0.722	8
Bayram	2016	Turkey	Caucasian	Gastric cancer	104	16	44	44	209	61	82	66	0.002	9
Li	2016	China	Asian	Lung cancer	391	109	201	81	337	117	159	61	0.587	9
Shiraishi	2016	Japan	Asian	Lung cancer	6830	2057	3386	1387	15155	5723	7133	2299	0.323	13
Wei	2015	China	Asian	Lung cancer	702	190	353	159	2520	814	1269	437	0.130	12
Shadrina 1	2015	Russia	Caucasian	Prostate cancer	360	102	183	75	358	105	165	88	0.150	11
Shadrina 2	2015	Russia	Caucasian	Breast cancer	642	192	310	140	523	132	280	111	0.097	12
Mosrati	2015	Sweden	Caucasian	AML	226	48	113	65	788	201	406	181	0.382	10
Liu	2015	China	Asian	Lung cancer	288	72	139	77	317	92	173	52	0.052	9
Du	2015	China	Asian	Gastric cancer	1105	360	557	188	994	346	464	184	0.197	11
de Martino	2015	Austria	Caucasian	RCC	241	61	120	60	375	97	181	97	0.502	10
Choi	2015	South Korea	Asian	Gastric cancer	243	34	107	102	246	38	122	86	0.625	8
Campa	2015	Germany	Caucasian	Pancreatic cancer	1724	445	861	418	3512	817	1763	932	0.764	13
Campa	2015	Germany	Caucasian	Multiple myeloma	2052	535	958	559	2633	634	1285	714	0.237	13
Adel Fahmideh	2015	Sweden	Caucasian	Brain tumor	240	61	103	76	478	109	256	113	0.120	12
Yin	2014	China	Asian	Lung cancer	524	139	273	112	524	186	255	83	0.777	11
Wang	2014	China	Asian	Lung cancer	1552	455	764	333	1605	549	780	276	0.971	12
Liorca-Cardenosa	2014	Spain	Caucasian	Melanoma	629	146	297	186	371	94	177	100	0.380	9
Zhao	2013	China	Asian	Lung cancer	1759	596	1163^a^	1163^a^	1804	674	1130^a^	1130^a^	/	9
Sheng	2013	China	Asian	ALL	569	178	270	121	656	233	323	100	0.490	13
Pellatt	2013	USA	Caucasian	Breast cancer	3698	1450	1934	314	3534	1179	1674	681	0.047	11
Pellatt 1	2013	USA	Caucasian	Colon cancer	1555	410	798	347	1956	493	956	507	0.321	12
Pellatt 2	2013	USA	Caucasian	Rectal cancer	754	214	356	184	959	270	465	224	0.386	12
Myneni	2013	China	Asian	Lung cancer	352	122	141	89	447	157	212	78	0.659	8
Ma	2013	China	Asian	Bladder Cancer	177	55	87	35	961	340	455	166	0.516	10
Lan	2013	China	Asian	Lung cancer	193	43	109	41	197	70	103	24	0.137	9
Wang	2012	China	Asian	Cervical Cancer	1010	322	462	226	1006	352	480	174	0.637	11
Rajaraman ^b^	2012	USA	Caucasian	Glioma	1854	/	/	/	4949	/	/	/	/	12
Kinnersley	2012	UK	Caucasian	Colorectal cancer	16039	4191	8105	3743	16430	4090	8082	4258	0.039	12
Ito	2012	Japan	Asian	Lung cancer	716	248	340	128	716	279	329	108	0.496	12
Hofer	2012	Austria	Caucasian	Colorectal cancer	137	38	68	31	1705	458	859	388	0.700	11
Chen	2012	China	Asian	Lung cancer	196	45	101	50	229	69	112	48	0.838	10
Shiraishi	2012	Japan	Asian	Lung cancer	4648	1386	2265	997	12364	4650	5856	1858	0.838	13
Bae	2012	Korea	Asian	Lung cancer	1094	402	501	191	1100	422	522	156	0.790	10
Pande ^b^	2011	USA	Caucasian	Lung cancer	1681	/	/	/	1235	/	/	/	/	10
Nan 1	2011	USA	Caucasian	Melanoma	210	55	91	64	831	215	399	217	0.252	11
Nan 2	2011	USA	Caucasian	SCC	277	57	125	95	831	215	399	217	0.252	11
Nan 3	2011	USA	Caucasian	BCC	274	68	116	90	831	215	399	217	0.252	11
Kohno	2011	Japan	Asian	Lung cancer	377	142	175	53	325	116	165	39	0.090	9
Hu	2011	China	Asian	Lung cancer	8559	2393	4294	1872	9378	3231	4533	1614	0.724	13
Ding	2011	China	Asian	HC	1269	428	633	208	1322	449	651	222	0.591	12
Chen	2011	China	Asian	Glioma	953	244	515	194	1036	334	542	160	0.014	10
Jaworowsk 1	2011	Poland	Caucasian	Lung cancer	855	247	403	205	844	263	425	156	0.494	11
Jaworowsk 2	2011	Poland	Caucasian	Bladder Cancer	431	134	216	81	439	134	226	79	0.335	10
Jaworowsk 3	2011	Poland	Caucasian	Laryngeal cancer	413	124	211	78	406	130	199	77	0.956	10
Gago-Dominguez 1	2011	USA	Caucasian	Bladder Cancer	471	86	239	146	547	127	262	158	0.361	11
Gago-Dominguez 2	2011	USA	Asian	Bladder Cancer	499	141	260	98	525	174	274	77	0.064	10
Wang	2010	UK	Caucasian	Lung cancer	239	42	115	82	553	136	259	158	0.146	8
Turnbull	2010	UK	Caucasian	TGCT	1588	520	767	301	7683	1904	3718	2061	0.005	10
Miki	2010	Japan	Asian	Lung cancer	2086	622	1048	416	1103	4093	5246	1695	0.835	13
Kohno	2010	Japan	Asian	Lung cancer	1656	488	796	372	968	373	460	135	0.719	13
Hsiung	2010	China	Asian	Lung cancer	2308	599	1187	522	2321	852	1132	337	0.211	12
Yoon	2010	Korea	Asian	Lung cancer	1425	467	696	262	3011	1187	1405	419	0.921	11
Truong 1	2010	France	Caucasian	Lung cancer	9126	1878	4526	2722	11812	2853	5817	3142	0.116	13
Truong 2	2010	France	Asian	Lung cancer	1686	538	836	312	2101	775	1014	312	0.506	12
Schoemaker	2010	UK	Caucasian	Glioma	216	30	114	72	241	54	127	60	0.397	9
Shete	2009	USA	Caucasian	Glioma	4344	781	2213	1350	6457	1623	3122	1712	0.008	11
Landi ^b^	2009	USA	Caucasian	Lung cancer	5739	/	/	/	5848	/	/	/	/	11
Jin	2009	China	Asian	Lung cancer	1212	353	627	232	1339	450	658	231	0.719	13
Wrensch	2009	USA	Caucasian	Glioma	691	95	354	242	3981	1021	1904	1056	0.006	12

Abbreviations: ESCC: esophageal squamous cell carcinoma; NC: nasopharyngeal carcinoma; UTUC: upper tract urothelial carcinomas; MPN: myeloproliferative neoplasms; CML: chronic myeloid leukemia; AML: acute myeloid leukemia; RCC: renal cell carcinoma; ALL: acute lymphoblastic leukemia; SCC: squamous cell carcinoma; BCC: basal cell carcinoma; HC: hepatocellular carcinoma; TGCT: testicular germ cell tumor; HWE: Hardy-Weinberg equilibrium

a: Number of cases and controls for TG and GG genotypers. b: The allele frequence in the three studies was provided to estimate the association under allele contrast model (G vs. T).

### Meta-analysis results

**Figure 2:**
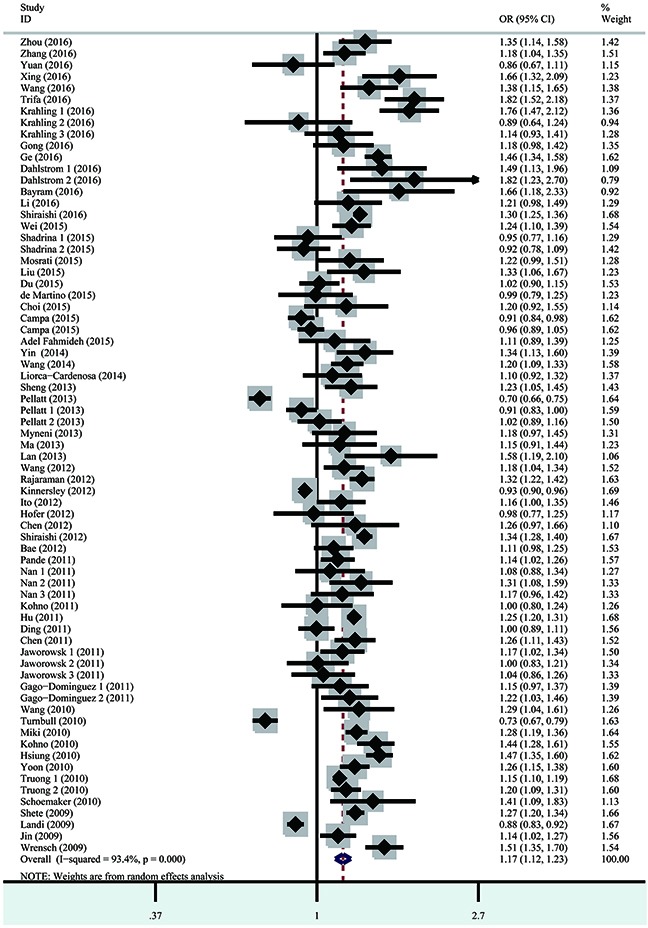
Forest plot of the association between the *TERT* rs2736100 polymorphism and overall cancer susceptibility in the allele contrast model

Heterogeneity among studies was observed for all five genetic models. Consequently, the random effect model was applied to calculate odds ratios (ORs). Risk estimates indicated that the *TERT* rs2736100 polymorphism was associated with overall cancer risk via all five genetic models [homozygous model (GG vs. TT): OR=1.39, 95% confidence interval (CI)=1.26–1.54, *P*<0.001; heterozygous model (TG vs. TT): OR=1.16, 95% CI=1.11–1.23, *P*<0.001; dominant model (TG + GG vs. TT): OR=1.23, 95% CI=1.15–1.31, *P*<0.001; recessive model (GG vs. TG + TT): OR=1.25, 95% CI=1.16–1.35, *P*<0.001; and allele contrast model (G vs. T): OR=1.17, 95% CI=1.12–1.23, *P*<0.001 (Figure 2, Table 2)]. The stratified analysis by cancer type associated the *TERT* rs2736100 polymorphism with lung cancer risk (homozygous model: OR=1.60, 95% CI=1.49–1.71, *P*<0.001; heterozygous model: OR=1.25, 95% CI=1.20–1.31, *P*=0.008; dominant model: OR=1.33, 95% CI 1.26–1.39, *P*<0.001; recessive model: OR=1.40, 95% CI=1.32–1.48, *P*<0.001; and allele contrast model: OR=1.24, 95% CI=1.17–1.31, *P*<0.001). This polymorphism was also associated with increased risk for thyroid cancer, bladder cancer, glioma, MPN and AML. Inversely, the *TERT* rs2736100 polymorphism was associated with decreased colorectal cancer risk (homozygous model: OR=0.86, 95% CI=0.82–0.91, *P*=0.512; dominant model: OR=0.94, 95% CI=0.90–0.98, *P*=0.970; recessive model: OR=0.88, 95% CI=0.82–0.96, *P*=0.279; and allele contrast model: OR=0.93, 95% CI=0.90–0.96, *P*=0.548). Stratified analysis was also performed by patient ethnicity, sample size of controls, and quality score. Elevated cancer risk was found among Asians in all five genetic models and among Caucasians under all five genetic models except for the recessive model. Our results also associated the *TERT* rs2736100 polymorphism with elevated overall cancer risk in all subgroups divided by sample size of controls and quality score in all the five genetic models.

**Table 2:** Meta-analysis of *TERT* rs2736100 T>G polymorphism on cancer risk

Variables	Homozygous			Heterozygous			Recessive			Dominant			Allele		
	GG vs. TT			TG vs. TT			GG vs. (TG + TT)			(TG +GG) vs. TT			G vs. T		
	OR (95% CI)	P^het^	I^2^ (%)	OR (95% CI)	P^het^	I^2^ (%)	OR (95% CI)	P^het^	I^2^ (%)	OR (95% CI)	P^het^	I^2^ (%)	OR (95% CI)	P^het^	I^2^ (%)
All	**1.39 (1.26-1.54)**	<0.001	93.3	**1.16 (1.11-1.23)**	<0.001	80.0	**1.25 (1.16-1.35)**	<0.001	91.1	**1.23 (1.15-1.31)**	<0.001	88.9	**1.17 (1.12-1.23)**	<0.001	93.4
Cancer type															
Lung	**1.60 (1.49-1.71)**	<0.001	65.7	**1.25 (1.20-1.31)**	0.008	45.5	**1.40 (1.32-1.48)**	<0.001	61.2	**1.33 (1.26-1.39)**	<0.001	58.6	**1.24 (1.17-1.31)**	<0.001	89.4
MPN	**3.17 (2.51-4.00)**	0.854	0.0	**2.03 (1.64-2.51)**	0.972	0.0	**1.89 (1.59-2.24)**	0.616	0.0	**2.40 (1.97-2.94)**	0.957	0.0	**1.74 (1.56-1.95)**	0.679	0.0
AML	**1.40 (1.04-1.88)**	0.631	0.0	1.22 (0.94-1.59)	0.744	0.0	1.23 (0.97-1.56)	0.411	0.0	**1.28 (1.00-1.64)**	0.970	0.0	**1.18 (1.02-1.37)**	0.658	0.0
Thyroid	**1.79 (1.25-2.56)**	0.076	68.3	1.26 (0.96-1.65)	0.085	66.2	**1.62 (1.37-1.92)**	0.266	19.3	**1.38 (1.02-1.88)**	0.041	76.0	**1.33 (1.08-1.64)**	0.040	76.4
Gastric	1.39 (0.82-2.33)	0.028	72.1	1.22 (0.90-1.66)	0.204	37.2	1.19 (0.83-1.70)	0.044	68.1	1.31 (0.90-1.90)	0.085	59.4	1.22 (0.94-1.58)	0.023	73.5
Breast	0.56 (0.25-1.28)	<0.001	95.0	0.88 (0.73-1.07)	0.158	49.8	0.63 (0.24-1.64)	<0.001	97.3	0.78 (0.71-0.85)	0.892	0.0	0.80 (0.61-1.04)	0.003	88.8
Melanoma	1.18 (0.90-1.54)	0.890	0.0	1.00 (0.78-1.27)	0.444	0.0	1.18 (0.95-1.47)	0.700	0.0	1.06 (0.85-1.33)	0.570	0.0	1.09 (0.95-1.26)	0.922	0.0
Colorectal	**0.86 (0.82-0.91)**	0.512	0.0	0.98 (0.93-1.03)	0.989	0.0	**0.88 (0.82-0.96)**	0.279	21.9	**0.94 (0.90-0.98)**	0.970	0.0	**0.93 (0.90-0.96)**	0.548	0.0
Bladder	**1.31 (1.08-1.59)**	0.481	0.0	1.15 (0.98-1.34)	0.498	0.0	**1.18 (1.00-1.39)**	0.598	0.0	**1.19 (1.02-1.38)**	0.436	0.0	**1.13 (1.03-1.25)**	0.507	0.0
Glioma	**1.89 (1.52-2.35)**	0.028	67.0	**1.55 (1.30-1.84)**	0.055	60.0	**1.35 (1.21-1.49)**	0.241	28.5	**1.65 (1.37-1.99)**	0.020	69.4	**1.33 (1.25-1.42)**	0.089	50.4
Others	1.09 (0.89-1.32)	<0.001	86.7	0.97 (0.88-1.07)	0.002	58.4	1.11 (0.95-1.29)	<0.001	84.3	1.01 (0.89-1.13)	<0.001	78.2	1.04 (0.94-1.15)	<0.001	87.0
Ethnicity															
Asian	**1.56 (1.46-1.67)**	<0.001	65.0	**1.22 (1.17-1.28)**	0.001	49.5	**1.39 (1.32-1.46)**	<0.001	50.4	**1.30 (1.28-1.36)**	<0.001	62.1	**1.25 (1.20-1.29)**	<0.001	67.7
Caucasian	**1.22 (1.04-1.44)**	<0.001	94.4	**1.12 (1.02-1.22)**	<0.001	83.6	1.11 (0.99-1.25)	<0.001	92.5	**1.16 (1.04-1.29)**	<0.001	90.7	**1.11 (1.03-1.19)**	<0.001	94.2
Sample Size															
≥ 500	**1.34 (1.19-1.51)**	<0.001	95.1	**1.16 (1.09-1.23)**	<0.001	83.7	**1.22 (1.11-1.33)**	<0.001	93.6	**1.21 (1.13-1.30)**	<0.001	91.5	**1.15 (1.09-1.22)**	<0.001	95.1
<500	**1.52 (1.26-1.82)**	<0.001	72.5	**1.19 (1.04-1.37)**	<0.001	66.2	**1.34 (1.19-1.51)**	0.001	55.1	**1.29 (1.11-1.49)**	<0.001	73.3	**1.23 (1.12-1.35)**	<0.001	74.1
Score															
High	**1.33 (1.18-1.48)**	<0.001	94.5	**1.15 (1.09-1.21)**	<0.001	82.7	**1.22 (1.12-1.33)**	<0.001	92.8	**1.20 (1.12-1.28)**	<0.001	90.8	**1.15 (1.09-1.21)**	<0.001	94.5
Low	**1.72 (1.40-2.10)**	0.001	60.5	**1.30 (1.10-1.54)**	0.003	57.0	**1.41 (1.26-1.59)**	0.154	27.4	**1.40 (1.20-1.63)**	<0.001	65.7	**1.30 (1.18-1.43)**	<0.001	60.5

Abbreviations: MPN, Myeloproliferative neoplasms; AML, Acute myeloid leukemia

### Heterogeneity and sensitivity analyses

Heterogeneity was detected amongst studies with respect to the association between the *TERT* rs2736100 polymorphism and overall cancer risk (homozygous model: *P*<0.001; heterozygous model: *P*<0.001; dominant model: *P*<0.001; recessive model: *P*<0.001; and allele contrast model: *P*<0.001). Therefore, we used the random effects model to generate pooled ORs and 95% CIs. Sensitivity analyses indicated that no single study could change the between-study heterogeneity and the results of meta-analysis.

### Publication bias

**Figure 3:**
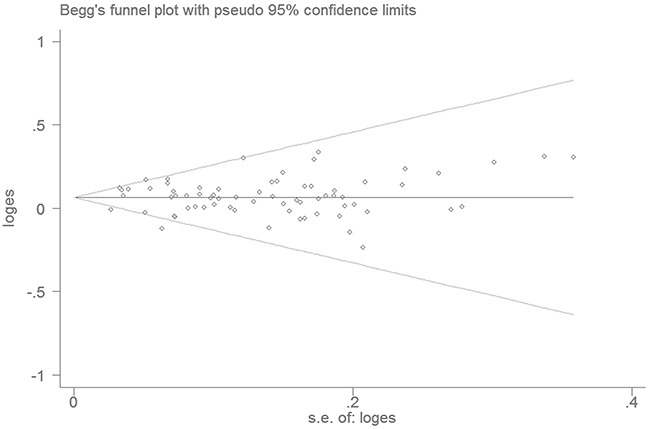
Funnel plot analysis to evaluate publication bias

The Begg's funnel plot and Egger's linear regression analysis did not reveal any evidence of publication bias in the meta-analysis (homozygous model: *P*=0.183; heterozygous model: *P*=0.805; dominant model: *P*=0.406; recessive model: *P*=0.085; and allele model: *P*=0.122; Figure 3).

### False positive report probability (FPRP) analyses

We calculated FPRP values for associations between the *TERT* rs2736100 T>G polymorphism and overall cancer risk using the five genetic models. FPRP values were all <0.20, suggesting that these associations were noteworthy (Table 3).

**Table 3:** False-positive report probability values for associations between the *TERT* rs2736100 T>G polymorphism and overall cancer risk

Genetic models	OR (95% CI)	*P*	Power	Prior Probability					
				0.25	0.1	0.01	0.001	0.0001	0.00001
Homozygous (GG vs. TT)	1.39 (1.26-1.54)	<0.001	0.555	0.000	0.000	0.000	0.000	0.000	0.000
Heterozygous (TG vs. TT)	1.16 (1.11-1.23)	<0.001	0.872	0.000	0.000	0.000	0.001	0.008	0.073
Recessive (GG vs. TG + TT)	1.25 (1.16-1.35)	<0.001	0.841	0.000	0.000	0.000	0.000	0.000	0.002
Dominant (TG +GG vs. TT)	1.23 (1.15-1.31)	<0.001	0.957	0.000	0.000	0.000	0.000	0.000	0.000
Allele (G vs. T)	1.17 (1.12-1.23)	<0.001	0.839	0.000	0.000	0.000	0.000	0.000	0.000

## DISCUSSION

Telomeres are special structures at the ends of eukaryotic chromosomes, and are responsible for protecting chromosomes from degradation, end-to-end fusion, and rearrangement [10]. Telomerase maintains proper telomere length by adding repetitive telomeric sequences to the 3′ ends of telomeres. Abnormal telomerase activity is implicated in the initiation and development of cancer and other age-associated diseases [75]. The TERT subunit of telomerase consists of three highly conserved domains: the RNA-binding domain (TRBD), the reverse transcriptase domain, and a carboxy-putative extension (CTE) proposed to constitute the putative thumb domain [75]. TERT is overexpressed in many human cancers [76]. The *TERT* rs2736100 polymorphism, localized in the second intron of the *TERT* gene, has been wildly studied with respect to cancer risk [7, 8]. However, the functional significance of the *TERT* rs2736100 polymorphism was not clear. Preliminary studies in gastric cancer suggested that this SNP is associated with decreased telomere length [9].

The present meta-analysis, comprising 108,248 cases and 161,472 controls, found that the *TERT* rs2736100 polymorphism increased overall cancer risk by 16–39%, suggesting that this SNP may contribute to carcinogenesis. A previous meta-analysis conducted by Zou, *et al*. in 2012 [15] also concluded that this polymorphism was associated with increased cancer risk. However, this analysis included only 11 case-control articles with 23,032 cases and 38,274 controls, which studied only lung cancer, glioma, and bladder cancer. Our stratified analysis by cancer type showed that the *TERT* rs2736100 polymorphism correlated with increased risk of lung cancer and glioma. Such associations were also observed in lung cancer- and glioma-specific meta-analyses published in 2014 [10–15, 77]. Between 2015 and 2016, at least 27 studies (6 studies on lung cancer) were published investigating the association between the *TERT* rs2736100 polymorphism and overall cancer susceptibility. To the best of our knowledge, ours is the largest meta-analysis of this association, with the strongest statistical power. Apart from lung cancer, glioma, and bladder cancer, our meta-analysis also investigated the association between the *TERT* rs2736100 polymorphism and risk of colorectal cancer (4 studies), MPN (4), gastric cancer (3), AML (2), breast cancer (2), melanoma (2), and thyroid cancer (2) as well as “other cancers” (16). We observed that this polymorphism was associated with decreased colorectal cancer risk. Since only four colorectal cancer studies were included in our meta-analysis, such an association might be a false positive, and validation will require further study.

The current meta-analysis had several limitations. First, there were substantial heterogeneities in the pooled study investigating the association between the *TERT* rs2736100 polymorphism and overall cancer risk. We reduced the degree of heterogeneity through stratified analyses by cancer type, patient ethnicity, sample size, and study quality score. Some cross-study heterogeneity might be attributed to differences among ethnic groups [78]. However, other sources of heterogeneity were not identified, such as control sources and genotyping methods. Second, the studies in this meta-analysis focused on Asian and Caucasian populations only, so we may not have had sufficient statistical power to evaluate associations based on ethnicity. Third, our results were based on unadjusted ORs due to the unavailability of confounding factor information for cases and controls (e.g., age, sex, smoking status, drinking status, and environmental exposure). Finally, lacking the original data from eligible studies limited our ability to explore gene-environment interactions.

In conclusion, our meta-analysis indicated that the *TERT* rs2736100 polymorphism was associated with increased overall cancer risk, especially lung cancer risk. Larger studies involving patients of different ethnicities are needed to confirm our findings.

## MATERIALS AND METHODS

### Identification of eligible studies

A comprehensive literature search of the PubMed and EMBASE databases was performed up to November 1, 2016. To find all eligible case-control studies that assessed the association between the *TERT* rs2736100 polymorphism and cancer risk, we used the following keywords: “TERT or telomerase reverse transcriptase”, “polymorphism or variant”, and “cancer or tumor or neoplasm or carcinoma”. We also evaluated additional studies by manually screening the references of both primary articles and reviews.

### Inclusion and exclusion criteria

Eligible studies included in our analysis met the following criteria: (i) the *TERT* rs2736100 polymorphism-cancer risk association was assessed; (ii) case-control studies or cohort studies; (iii) sufficient data to calculate an OR with 95% CI; (iv) studies in English. Exclusion criteria were as follows: (i) case only studies; (ii) overlapping publications; (iii) abstract, case report, editorial comment, and review. Studies that deviated from HWE in controls were excluded, unless further evidence showed that another polymorphism was in HWE.

### Data extraction

Two investigators independently extracted available data from each eligible study. The following information was collected: first author's surname, year of publication, country of origin, patient ethnicity, cancer type, numbers of cases and controls, genotype counts of cases and controls, results of the HWE test, and quality scores (low quality studies with score ≤9, high quality studies with score >9) [79]. Any disagreements were solved by discussion until a consensus was reached between the two investigators. If no consensus was reached, another investigator joined the discussion, and a final decision was made by a majority.

### FPRP analysis

FPRP values were applied to assess the statistical power of our significant findings [80, 81]. An FPRP value of 0.20 was set as the criterion for noteworthiness. A prior probability of 0.1 was assigned to detect an OR of 0.67/1.50 (protective/risk effects) for an association with genotypes under investigation.

### Statistical analysis

HWE in control subjects was assessed by chi-squared test. The strength of association between the *TERT* rs2736100 polymorphism and cancer risk was estimated by calculating crude ORs and their 95% CIs using all five genetic models: homozygous (GG vs. TT), heterozygous (TG vs. TT), dominant (GG vs. TG + TT), and recessive (TG + GG vs. TT), as well as the allele contrast model (G vs. T). Q-test was used to quantify heterogeneity among all eligible studies, and *P*>0.10 suggested a lack of heterogeneity among studies. Generally, the fixed effects model (Mantel–Haenszel method) or the random effects model (DerSimonian–Laird method) was employed in the absence (*P*≥0.10) or presence (*P*<0.10) of heterogeneity, respectively [82–84]. Heterogeneity was also estimated using the I^2^ test [85]. Subgroup analyses were conducted by patient ethnicity, cancer type, and study sample size. The Begg's funnel plot and the Egger's linear regression test were used to evaluate publication bias [86]. All statistical analyses were performed using STATA version 12.0 software (STATA Corporation, College Station, TX). All statistical analyses were two-sided. P<0.05 was considered statistically significant.
